# Manual of Diseases of Women

**Published:** 1885-12

**Authors:** 


MANUAL OF THE DISEASES OF WOMEN, being a concise and systematic exposition of the theory and practice of Gynæcology, for use of students and practitioners. By Charles W. May, M. D., late Home Physician Mt. Sinai Hospital, New York; Assistant to the Chair of Ophthalmology, New York Polyclinic; Clinical Assistant Department of Ophthalmology Manhattan Eye and Ear Hospital, New York. Philadelphia: Lea Brothers & Co. 1885.

This neatly printed volume of 357 pages contains an exposition of the theory and practice of the diseases peculiar to women, and these have been condensed, classified and arranged so as to make the book a short and systematic treatise, which may be useful to the practitioner who wishes to refresh his memory touching female disorders.

